# Hotspots and trends in gastric cancer stem cell research: a visualization and bibliometric analysis

**DOI:** 10.3389/fonc.2025.1523465

**Published:** 2025-03-05

**Authors:** Jinfeng Liu, Xinhui Han, Qingyi Wang, Sihui Qin, Yujie Xi, Guoying Liang

**Affiliations:** ^1^ School of Clinical Medicine, Heilongjiang University of Chinese Medicine, Harbin, China; ^2^ Department of Gastroenterology 1, The First Affiliated Hospital of Heilongjiang University of Chinese Medicine, Harbin, China

**Keywords:** CiteSpace, VOSviewer, gastric cancer stem cells, bibliometric analysis, visualization

## Abstract

**Background:**

Gastric cancer (GC) is a type of malignant tumor that seriously endangers human health. As the understanding of the mechanisms underlying gastric cancer deepens, in recent years, investigations on gastric cancer stem cells (GCSCs) have garnered significant interest. They are pivotal in the onset, progression, recurrence, and pharmacoresistance of GC. Comprehensive research on GCSCs is expected to provide new strategies for the diagnosis and treatment of GC. This article endeavors to comprehensively assess the current status and future trends of GCSCs research through bibliometric analysis, thereby providing a valuable reference for further in - depth studies in this field.

**Methods:**

English - language academic journals related to GCSCs research in the Web of Science database were retrieved. Subsequently, VOSviewer was utilized to conduct network collinear analysis of the exported source institutions, literature authors, references, and keywords. And CiteSpace was used to perform statistical analysis of the annual publication count, keyword clustering, references, and keyword burst.

**Results:**

A total of 3882 documents that met the criteria were incorporated. The quantity of published papers has shown a consistent upward trend annually since 2003. Among the authors of the literature, multiple stable core author groups represented by Zhu, Wei, Wang, Mei, Xu, Wenrong, etc. have been formed. There are 335 associated institutions in total. The Japan National Cancer Center has the strongest relevance and the largest number of published papers. There are 7 clustering labels formed among the keywords, including main clustering modules such as activation, cancer stem cells, DNA content aneuploidy, and expression. 25 burst keywords were generated, and the burst keywords in the past two years include mesenchymal stem cells, drug resistance, proliferation, etc. The emergence of references indicates that eight references have been cited up to now and are the focus of current research.

**Conclusion:**

The research overview of GCSCs in the past 30 years was visually presented by visual maps. In the past decade, scholars’ research in this field has gradually intensified, and the development trend is good. Through the deeper study of the GCSCs mechanism, intervention GCSCs in the future will be a new promising treatment approach for GC patients. This hot topic still deserves more attention in the future.

## 1 Introduction

Recent data from the World Health Organization indicates that stomach cancer ranks fifth in the world in terms of diagnosed cancer cases and is the fourth leading cause of cancer deaths (1). The latest progress in cancer stem cell research has significantly improved the level of cancer treatment (2). And the study of gastric cancer stem cells (GCSCs) has become a central focus of gastric cancer therapy. GCSCs derive from stomach adult stem cells and bone marrow cells. These cells can produce several types of gastric mucosal cells and are essential for gastric mucosal homeostasis. They are crucial for the repair of stomach mucosal injuries and the dynamic renewal of the epithelium. Moreover, they are involved in the development, progression and recurrence of gastric cancer (3). Consequently, examining the characterization of GCSCs aids in the accurate identification of targets for potential therapeutic approaches for gastric cancer and is crucial for the targeted therapy and prognosis of the disease.

To elucidate the knowledge structures and developmental trajectories within a field of study, bibliometric analysis has advanced into a refined statistical methodology that uses quantitative techniques to amass substantial bibliometric data using developed algorithms (4). Researchers can get an intuitive comprehension of the literature’s characteristics, study focal points, and developmental trajectories in this subject by constructing networks of multi-tiered research elements (including institutions, countries, and keywords) (5). For this reason, the author uses CiteSpace and VOSviewer software to visually analyze the research hotspots and trends in the field of GCSCs since the establishment of the database, providing a certain reference for the research ideas and directions of scholars in this field.

## 2 Materials and methods

### 2.1 Literature collection

**Figure 1:**
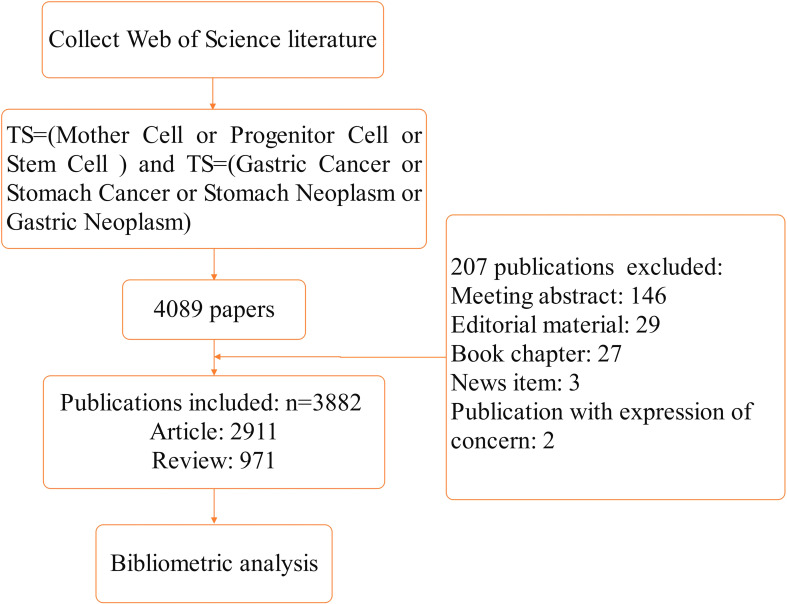
Flow chart of literature screening.

Articles in the WoS core database were retrieved using the subject words “TS= (Mother Cell or Progenitor Cell or Stem Cell) and TS= (Gastric Cancer or Stomach Neoplasm or Gastric Neoplasm or Stomach Cancer)”. The language was set to English, and the search commencement time was unspecified. The end time was set to October 2024. Finally, 4089 articles were retrieved. The literature was imported into the Endnote literature management software in text format. Firstly, duplicate literature was checked. After deduplication, the information such as the author, institution, and keywords of the literature was checked for completeness. According to the content of the literature title and abstract, it was judged whether it conformed to the theme. Literature with incomplete content and not conforming to the theme was removed. Finally, 3882 pieces of literature were included as the data source for this visual analysis (**Figure 1**).

### 2.2 Data analysis

First, import 3,882 key documents into VOSviewer (1.6.18). Conduct network co-line analysis by selecting source institutions, countries, document authors, references, and keywords, respectively. Then export the document of the number of publications by country to prepare to create a co-line graph of the countries of document origin in Scimago Graphica, with relevant parameters set as default. Use CiteSpace (v.6.3.R1) to analyze keyword clustering, reference bursts and keyword bursts. Select “WOS” as the data source; Time span: 1994 – 2024, Slice Length = 5, Selection Criteria: Top 20 per slice, LRF = 3.0, L/N = 10, LBY = 5, e = 1.0. Select the number of publications, keyword clustering, references, and keywords as node types, respectively, and keep other parameter settings consistent with the system default. Use Office16 Excel to generate a scatter plot of the annual publication count.

## 3 Results

### 3.1 Analysis of the annual number of published literature

**Figure 2:**
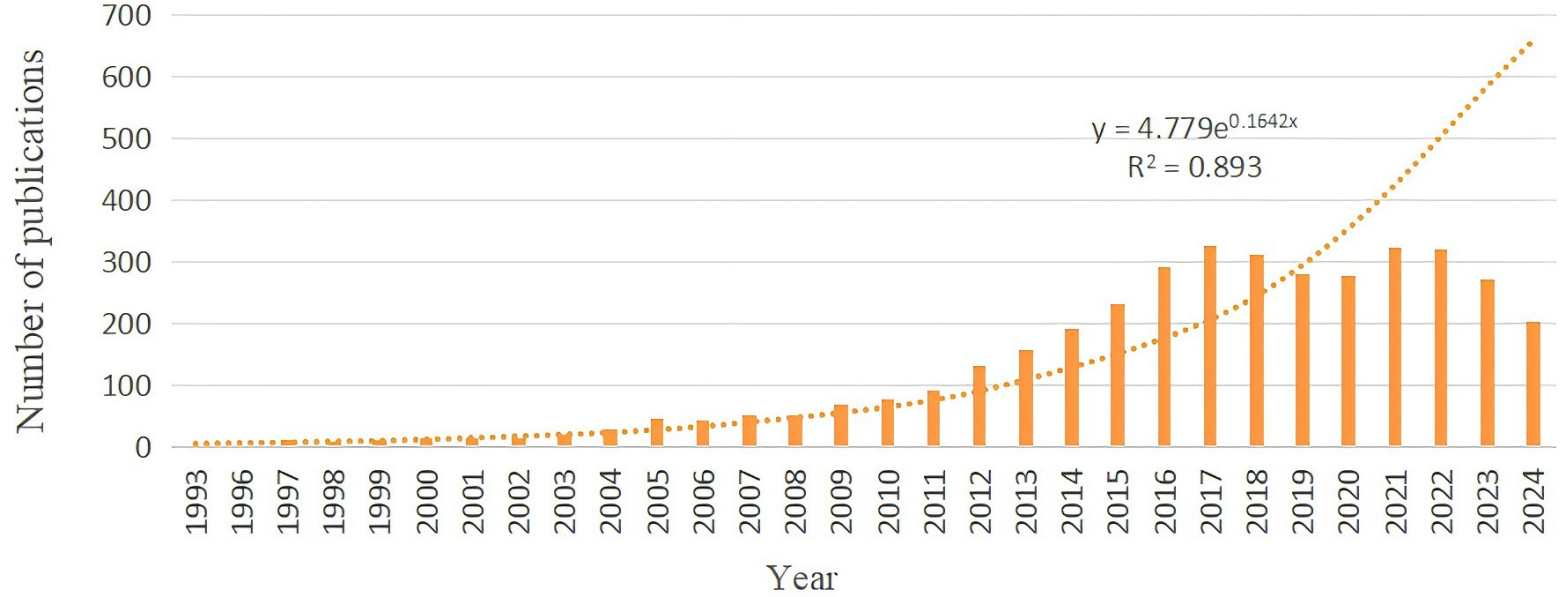
Annual number of publications in.

According to the analysis of literature retrieval results, from 2003 to 2022, the average annual number of published papers increased incrementally. As can be seen from the exponential trend in **Figure 2**, the number of papers published has shown an increasing trend year by year since 2003 (y = 4.779e^0.1642x^ R^2^ = 0.893, Among them, R²=0.893> 0.8, this exponential trend model shows a good fitting degree). The rapid growth of global published papers is largely attributed to the rapid development of the GCSCs research field. The continuous growth trend indicates that the future development and clinical application of this field have huge potential, and research output is continuously accumulating. In 2017, the number of globally published papers reached its peak (325 papers), attracting wide attention from scholars, Subsequently, the number of papers published continued to be stable.

### 3.2 Collinear analysis of literature author networks

**Figure 3:**
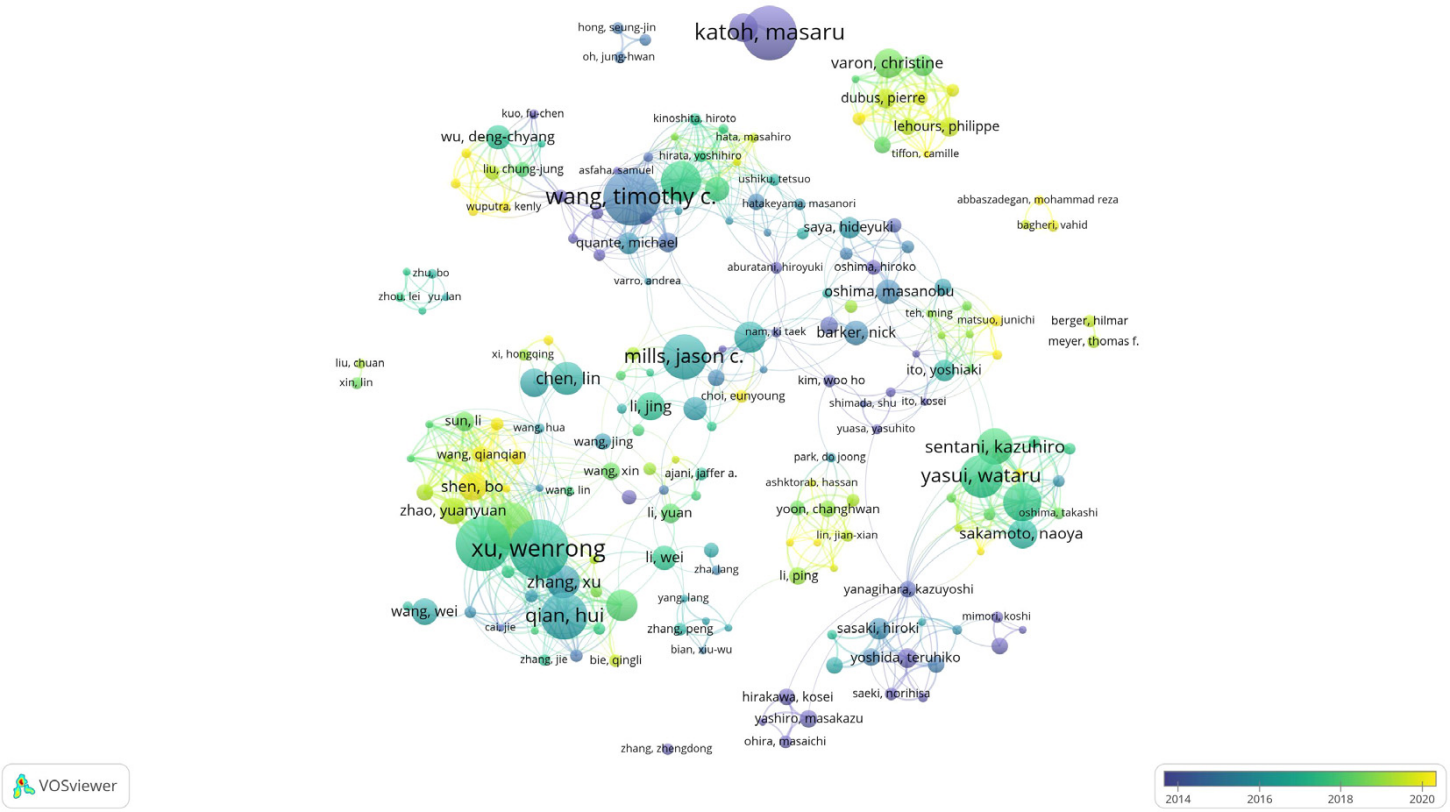
Co-authorship network collinear diagram.

According to the statistics of VOSviewer, there are 19,080 literature authors in total. Among them, katoh, masaru has published the largest number of papers, with a total of 36 papers. According to Price’s law, M = 0.749 √Nmax (Nmax represents the number of papers published by the author with the largest number of papers, and M represents the minimum number of papers). Authors with the number of papers above M are core authors. The result of this study is that M≈4.5. Therefore, the parameter number of papers published is set to ≥5. A total of 366 core authors are counted. The core authors are drawn into a network collinear diagram (**Figure 2**). As can be seen from the diagram, in this research field, multiple stable core author groups represented by xu, wenrong, wang, timothy c, zhu, wei and others have been formed. According to the graphic color, it can be seen that the teams of Zhu, Wei, Wang, Mei, Xu, Wenrong and others have strong relevance, and the time of publishing papers tends to be the present (**Figure 3**). **Table 1** shows the information on the number of publications of the top 10 authors.

**Table 1:** The top 10 authors on the research of GCSCs.

Rank	Author	Documents	Avg.pub.year	Link strength	Citations
1	Xu, Wenrong	36	2016	194	1435
2	Wang, Timothy C	34	2015	71	3513
3	Zhu, Wei	33	2017	190	1076
4	Katoh, Masaru	33	2010	22	3036
5	Katoh, M	29	2004	11	1271
6	Wang, Mei	28	2018	191	829
7	Qian, Hui	28	2016	131	1287
8	Mills, Jason C.	27	2016	28	1339
9	Yasui, Wataru	26	2017	117	760
10	Hayakawa, Yoku	24	2017	91	1201

### 3.3 Collinear analysis of literature research institution networks

**Figure 4:**
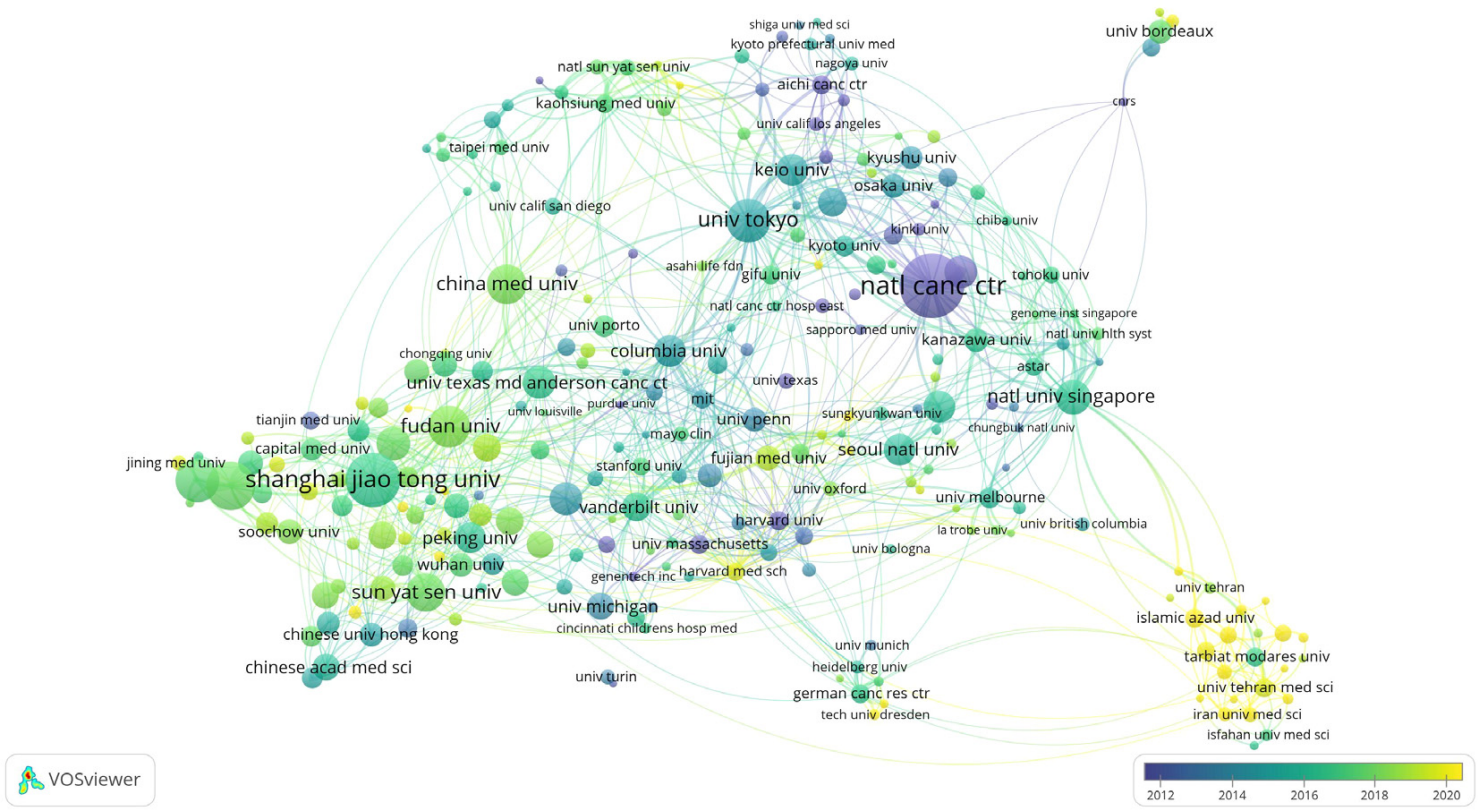
Institutional cooperation network collinear diagram.

According to the statistics of VOSviewer, there are a total of 335 institutions with a publication volume of ≥5. According to the publication volume ranking, **Table 2** summarizes the publication information of the top 10 scientific research institutions. Seven are Chinese institutions, two are Japanese institutions, and one is a Singaporean institution. Among them, the Japanese institution Japan National Cancer Center has the highest number of publications. Its citation number is significantly higher, indicating that its influence is relatively high (**Figure 4**).

**Table 2:** The top 10 institutions on GCSCs.

Rank	Institution	Number of papers	Average year	Correlation strength	Citations
1	Japan National Cancer Center	137	2011	161	8083
2	Shanghai Jiao Tong University	103	2016	78	3094
3	Nanjing Medical University	87	2017	81	2447
4	Jiangsu University	73	2017	54	2919
5	University of Tokyo	71	2014	141	4821
6	Fudan University	67	2018	80	1746
7	China Medical University	63	2017	41	1530
8	Sun Yat-sen University	60	2017	53	2322
9	National Universityof Singapore	49	2016	126	3482
10	Zhejiang University	47	2017	26	895

### 3.4 Collinear analysis of national networks in literature research

**Figure 5:**
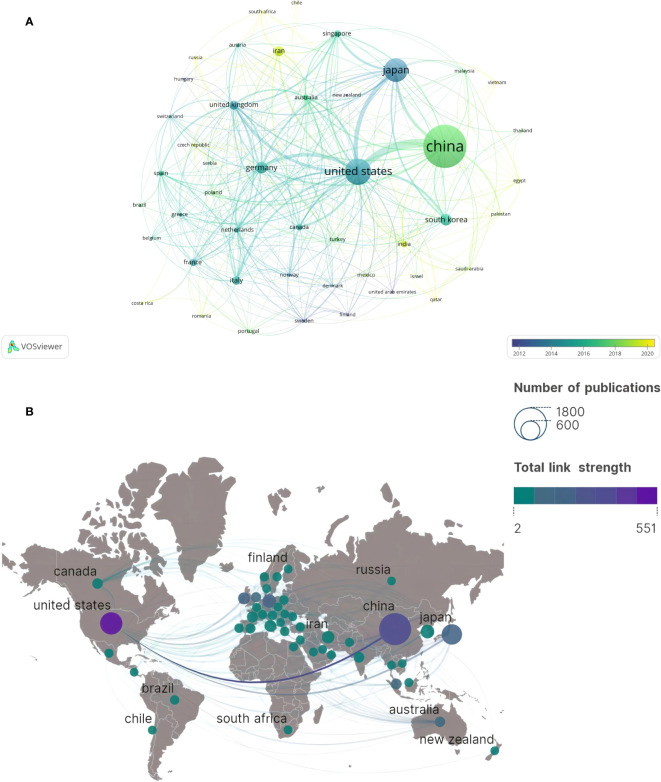
Country/region cooperation network collinear diagram. **(A)** Country/region collaboration map generated by VOSviewer. **(B)** Country/region collaboration map generated by Scimago Graphica.

A total of 76 countries or regions have published pertinent content, with 46 countries having five or more articles according to VOSviewer. **Table 3** encapsulates the data of the top 10 countries or regions, revealing that the aggregate citations of the leading three countries/regions much exceed those of other entities. **Figure 5** illustrates the cooperation network among these countries/regions, highlighting the extensive collaboration of the United States with several nations, particularly its primary partners, China and Japan.

**Table 3:** The top 10 countries on GCSCs.

Rank	Country	Number of papers	Average year	Correlation strength	Citations
1	China	1576	2017	330	43405
2	United States	700	2014	551	44950
3	Japan	571	2013	212	27000
4	South Korea	184	2016	78	4886
5	Germany	183	2015	182	8998
6	Iran	136	2019	60	3324
7	United Kingdom	117	2014	167	7691
8	Italy	107	2015	76	3615
9	France	79	2014	58	3482
10	Singapore	71	2016	100	3482

### 3.5 Keyword analysis

#### 3.5.1 Collinear network analysis

**Figure 6:**
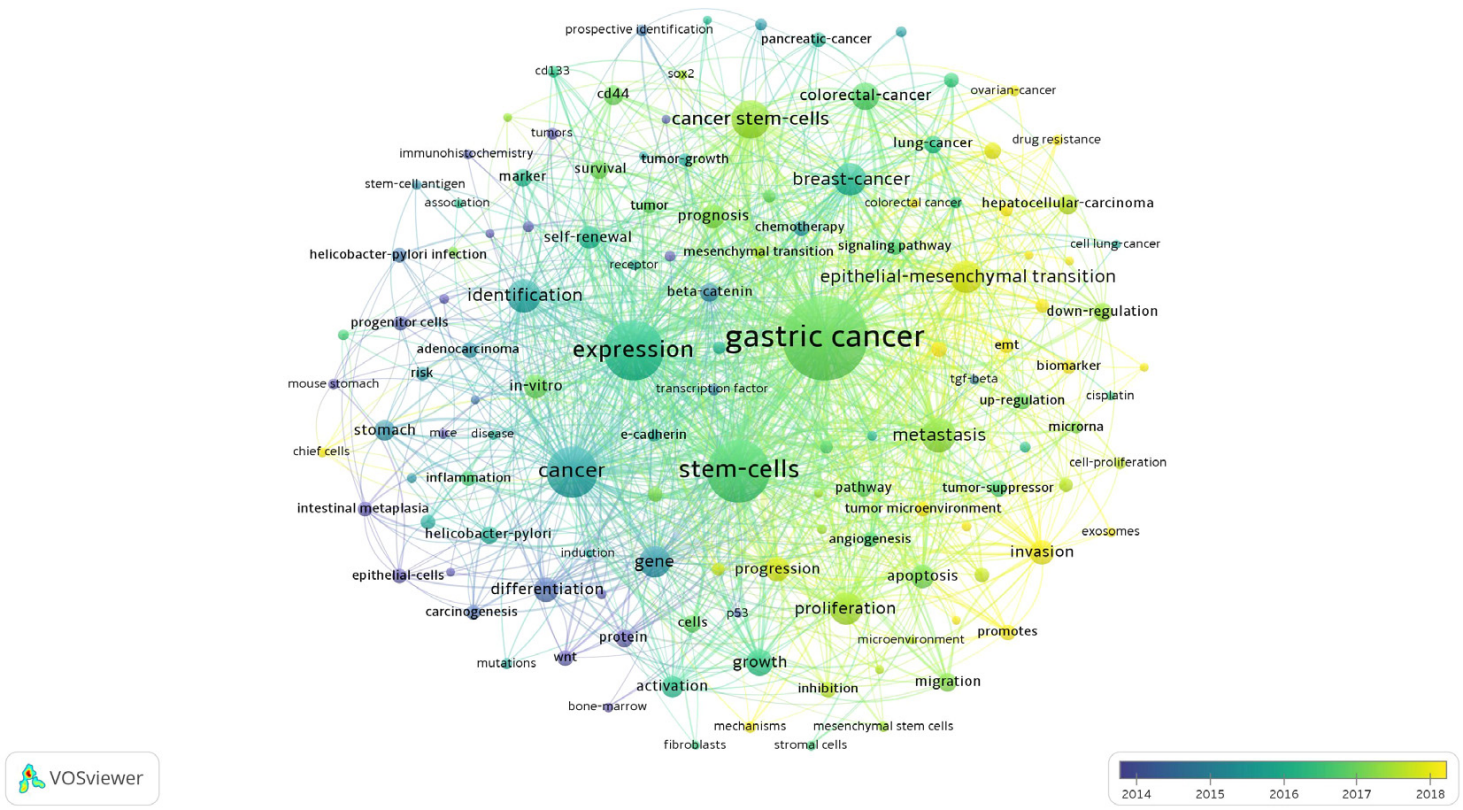
Network of keyword on GCSCs.

High-frequency keywords are important markers that reflect the dynamic development of the research frontiers in a particular field of knowledge. VOSviewer was used to draw a collinear network diagram. As shown in **Figure 6**, the larger the circular area, the greater the frequency of keyword occurrence. **Supplementary Table 1** lists the top 15 keywords ranked by frequency and centrality. They include “gastric cancer”, “stem cells”, “expression”, “cancer stem cells”, “breast cancer”, “identification”, “colorectal cancer”, “carcinoma”, “metastasis”, “epithelial mesenchymal transition”, “cancer”, “proliferation”, “growth”, “helicobacter pylori”, “*in vitro*”. The greater the frequency of keyword occurrence, the more widely it is studied. The greater the centrality of a keyword, the greater its influence in research. The centrality of “gastric cancer”, “lung cancer”, “carcinoma”, “cancer”, “stem cells”, “expression”, “helicobacter pylori”, “protein”, “differentiation”, “breast cancer”, “gene expression”, “gene”, “up-regulation”, and “growth” is all >0.1, indicating that this node is a central node. They are important research topics in this research field and have high influence (**Figure 5** **).**

#### 3.5.2 Cluster analysis

**Figure 7:**
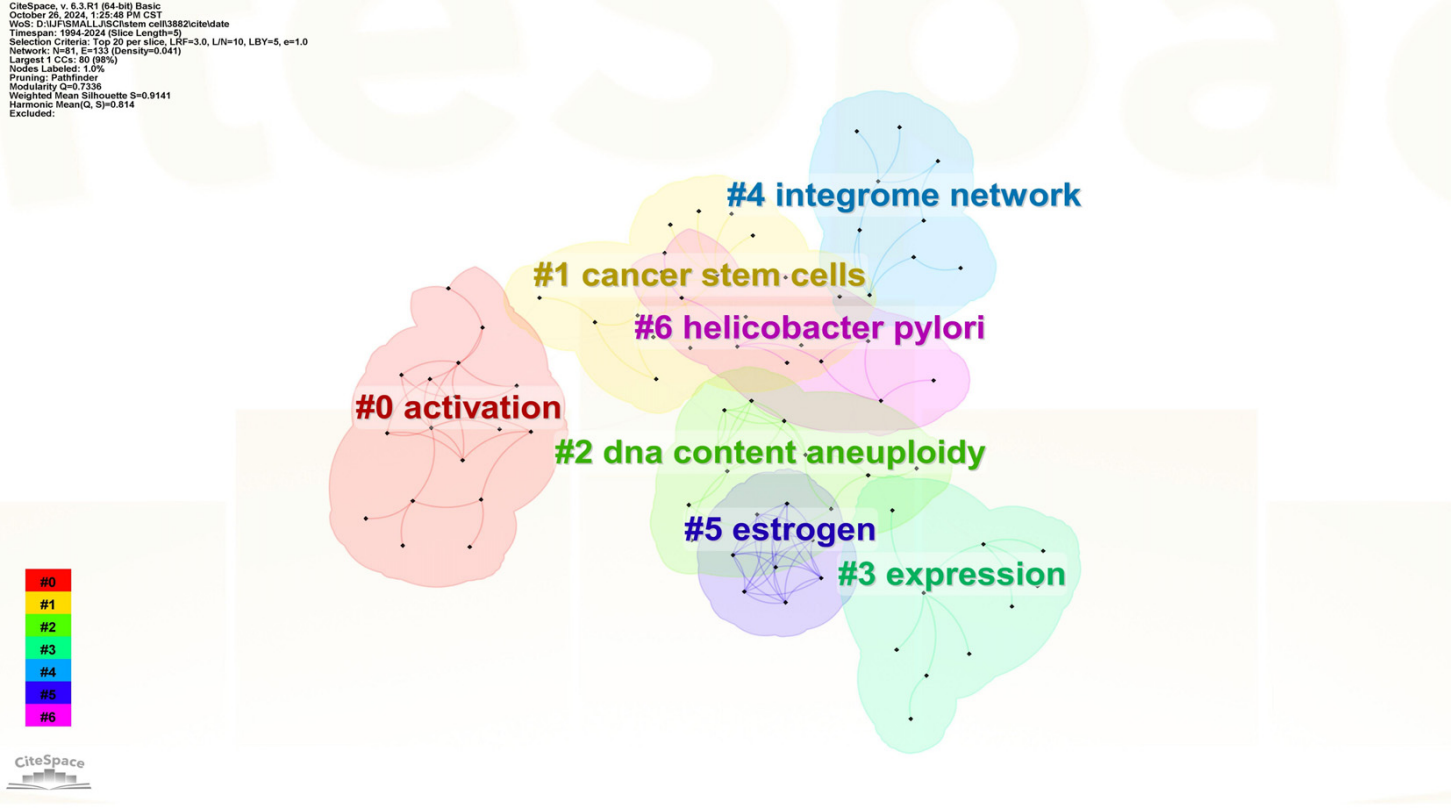
Keyword clustering on GCSCs.

The Log-likelihood rate algorithm in CiteSpace is employed for keyword cluster analysis. In **Figure 7**, the modularity Q value of the clustering is used to evaluate the structure of the atlas. The Q value ranges from (0, 1). Generally, it is considered that when Q > 0.3, the clustering structure is meaningful. The weighted average silhouette value S of clustering represents the clarity of clustering. When S > 0.5, the clustering is justifiable. When S > 0.7, the clustering result is convincing. In this study, Q = 0.7336 and S = 0.9141, indicating that this clustering is reasonable and convincing. It includes seven clustering modules: “#0 activation, #1 cancer stem cells, #2 DNA content aneuploidy, #3 expression, #4 integrome network, #5 estrogen, #6 Helicobacter pylori”.

The clustering module summarizes the factors involved in the development of GCSCs, with #0, #3 reflecting mutational factors, #5, #6 microenvironmental factors, #2, #4 epigenetic regulatory factors, and #1 reflecting the intrinsic characteristics of GCSCs, which are the main areas of relevance addressed in the current study.

#### 3.5.3 Keyword clustering time view

**Figure 8:**
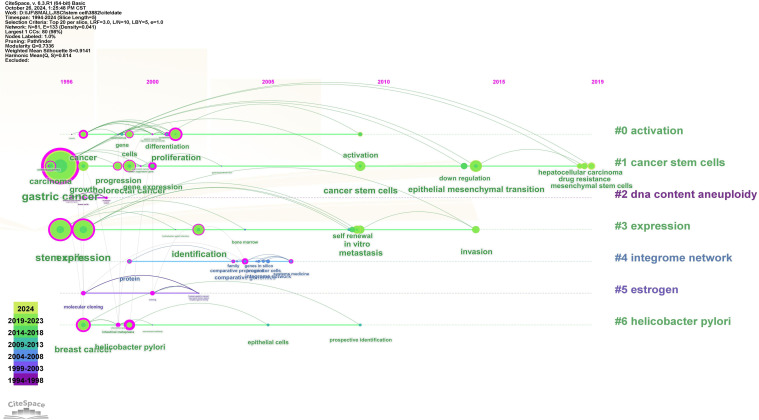
Keyword clustering time view.

The timeline view reflects the evolution and development of the theme of GCSCs over the past 30 years in terms of time. The appearance and disappearance of clustering keywords have certain characteristics of the times, and the research hotspots that scholars focus on in different time periods are different. Through the timeline view, keywords can be divided according to time, and the historical evolution process of the research theme can be found more intuitively. As shown in **Figure 8**, each node represents a keyword. The larger the node is, the more times the keyword has been studied. The connection line between two nodes indicates that there is a certain connection between them, and the more connection lines a node has, the stronger its connectivity is.

#### 3.5.4 Emergence analysis

**Figure 9:**
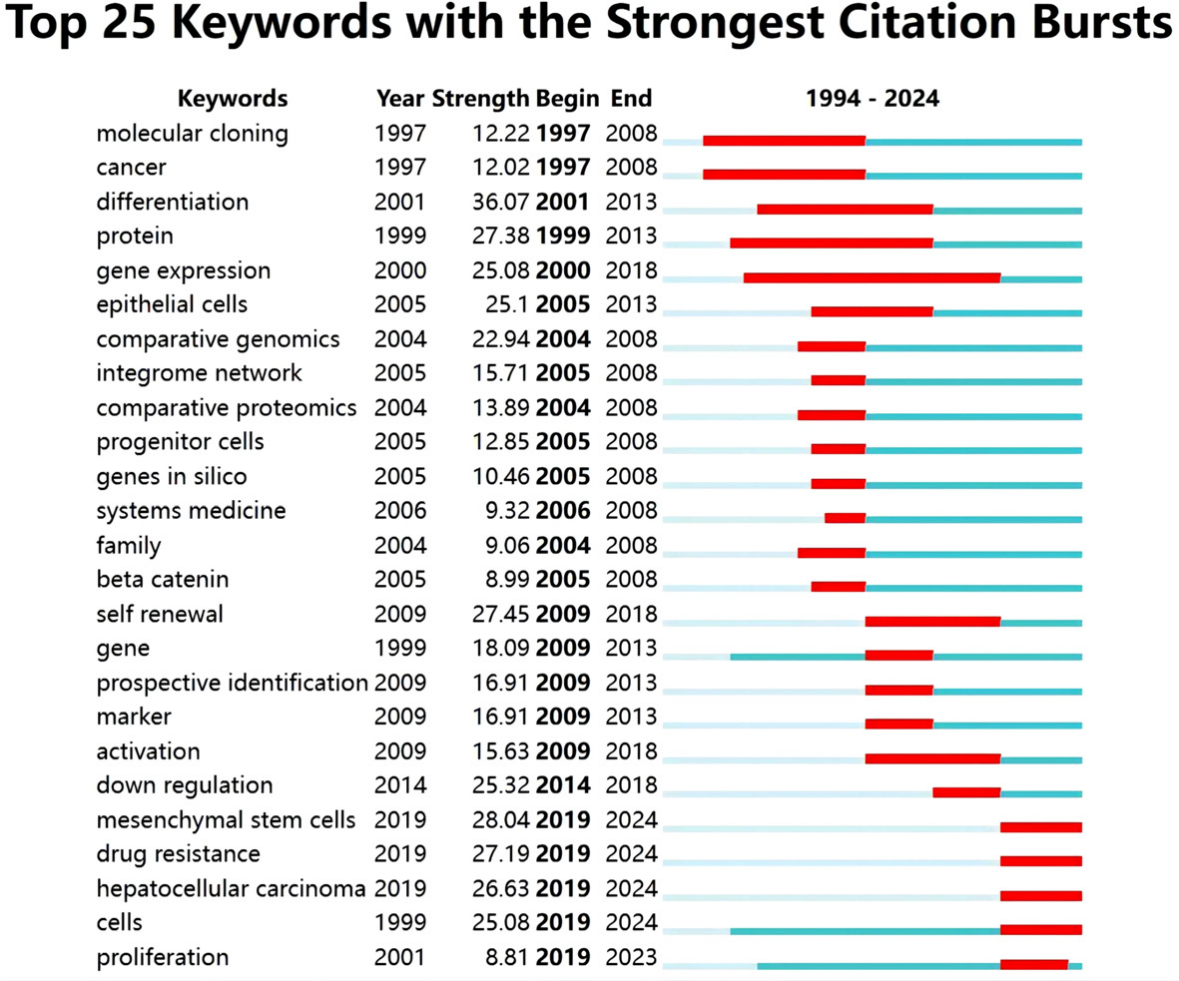
Top 25 Keywords with the strongest citation bursts.

Keyword burst detection can more accurately identify keywords that are popular and then decline in a certain period of time. A red line signifies that the associated terms commonly occurred during this period, whereas a blue line denotes their relatively occasional usage (**Figure 9**). Among the top 25 keywords in the picture with the strongest citation bursts: “molecular cloning” started in 1997 and ended in 2008. “cancer” had a similar pattern starting in 1997 and ending in 2008. differentiation’ was the keyword with the strongest burst (strength = 36.07), which reflects that it once had relatively high research value and influence (“protein”, “gene expression”, “epithelial cells”, and “self renewal”) had a duration not less than 5 years. Notably, the keywords of “mesenchymal stem cells” (strength=28.04), “drug resistance” (strength=27.19), “hepatocellular carcinoma” (strength=26.63)and “cells” (strength=25.08) have continued until 2024, may serve as a focal point for forthcoming study.

### 3.6 Analysis of co-cited references

**Figure 10:**
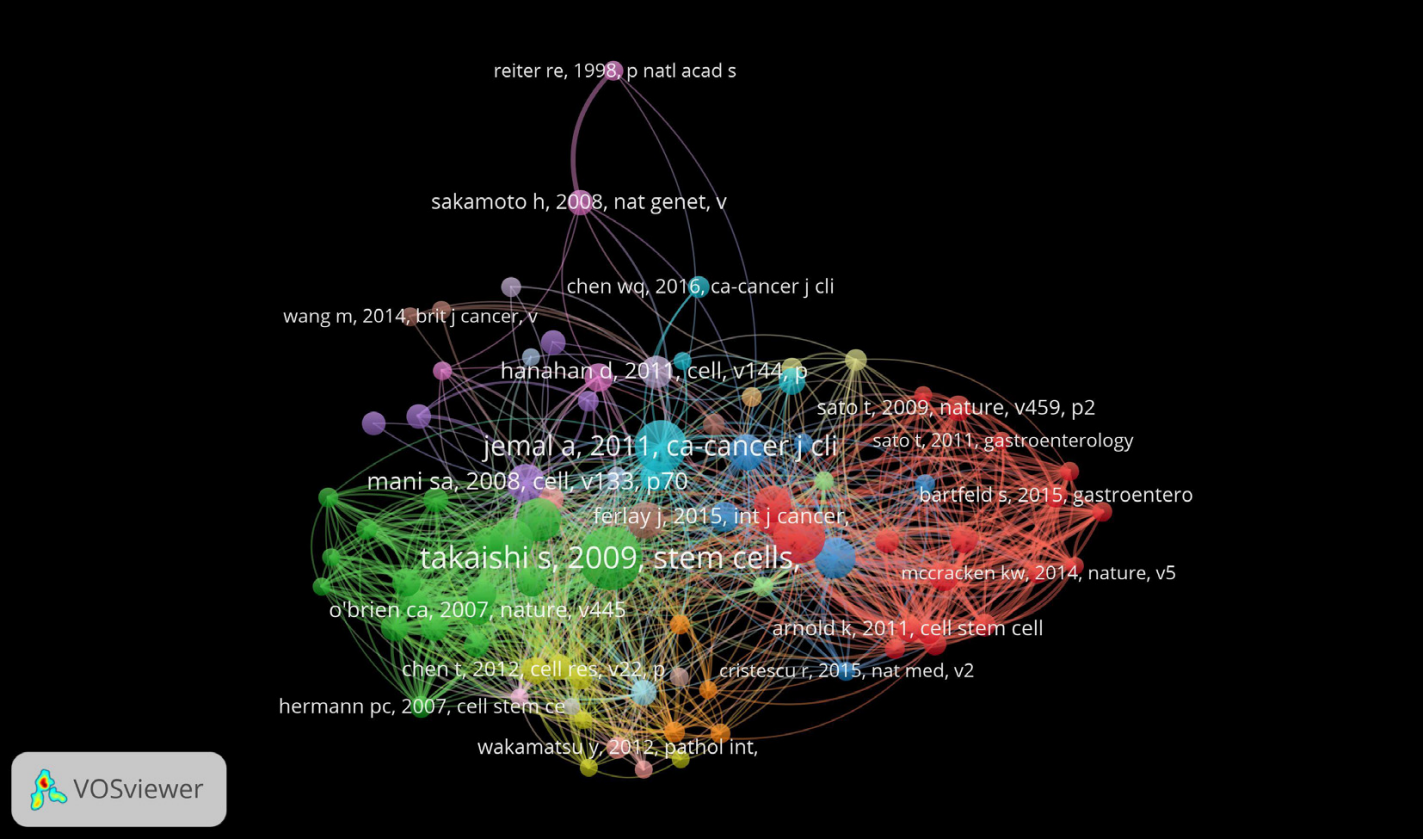
Network of co-cited references on research of GCSCs.

Co-cited references are treated as two simultaneous citations. 144,645 co-cited references are available for gastric cancer stem cell related studies from 2010 to 2024, of which 87 studies have more than 50 citations. **Supplementary Table 2** shows the top 10 most cited co-cited references. “Identification of gastric cancer stem cells using the cell surface marker CD44” (6) in Stem Cells had the highest number of citations (n = 380), followed by “Lgr5 (+ve) stem cells drive self - renewal in the stomach and build long - lived gastric units *in vitro*” (7) in Cell Stem Cell, followed by “Global cancer statistics” (8) in CA Cancer J Clin. A co-cited reference network mapping was also constructed for references with ≥50 co-citations (**Figure 10** **).**

### 3.7 Co - cited reference burst

**Figure 11:**
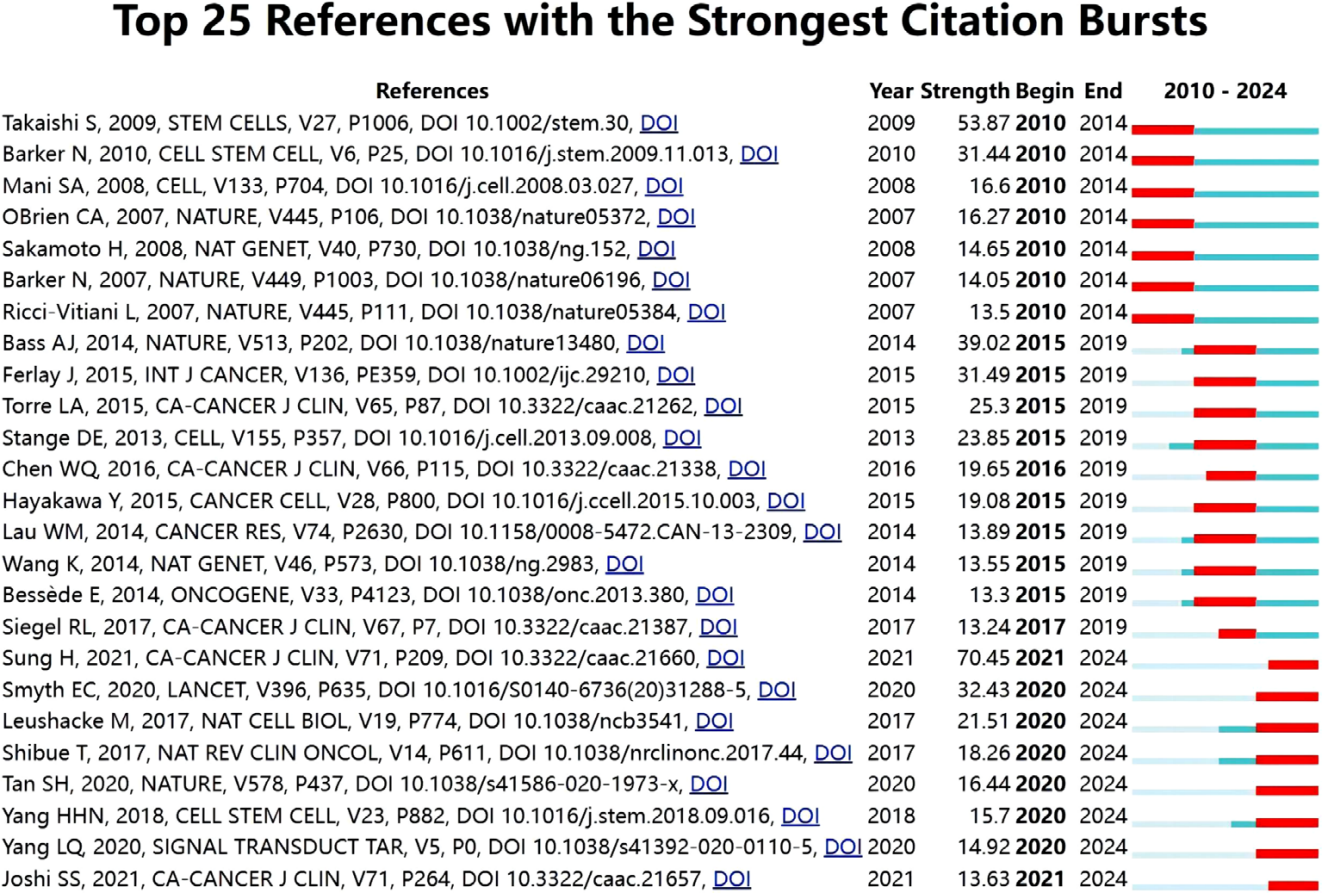
Top 25 references with the strongest citation bursts for publications on GCSCs.

Citation burst is a sharp increase in the frequency of publications over a certain period of time, which in part reflects the dynamics of a field. In the current study, **Figure 11** illustrates the top 25 references with the highest citation burst rate as determined by CiteSpace. As can be seen in **Figure 11**, Citation bursts occurred as early as 2009, and the intensity of these 25 references ranged from 13.3 to 70.45. Sung et al. in CA Cancer J Clin, “Global Cancer Statistics 2020: GLOBOCAN Estimates of Incidence and Mortality Worldwide for 36 Cancers in 185 Countries” (1), which had the highest citation burst rate of 70.45, has been cited from 2021 to the present. “Identification of gastric cancer stem cells using the cell surface marker CD44” (6), by Takaishi et al, also had a high citation burst intensity (intensity = 53.87), although the end year was relatively early.

## 4 Discussion

A century ago, European pathologists made a groundbreaking discovery that a tumor is a heterogeneous, incompletely differentiated population of cells. In the 1990s, the theory of tumor stem cells was proposed, providing a new theoretical basis for the origin, evolution, and formation of chemical drug resistance of tumors (9, 10). In 1994, CSCs were initially confirmed in human acute myeloid leukemia (11). Since this milestone, stem cells have been discovered in various tumor types. It was not until 2009 that Takaishi S et al. first reported the relationship between CD44 and GCSCs in six gastric cancer cell lines (6), and research on GCSCs gradually emerged. In the early stage, the team centered on Masaru Katoh began to study the signaling pathway network of gastrointestinal cancer stem cells, including WNT, noth, bmp, hedgehog and other signaling pathways (12, 13). In recent years, the team centered on Wenrong Xu has studied the relationship between GCSCs and exosomes, microRNAs, and gastric stromal stem cells (14, 15).

Tumor stem cells are a special class of subpopulations that are capable of both self - renewal and generation of multiple tumor cells in tumors. These cells can further differentiate to generate new tumor cells and are considered crucial in tumor genesis, dissemination, maintenance, metastasis, recurrence, and resistance to chemotherapy. GCSCs arise from normal gastric stem cells, progenitor cells, or differentiated cells that acquire self - renewal capabilities following mutations. A cohort of adult stem cells resides in the stomach isthmus and antrum isthmus, as well as in the regions beneath. They possess two fundamental characteristics: self - renewal and tissue renewal. The former can produce a greater number of undifferentiated gastric stem cells to maintain their presence in gastric mucosal tissue, whereas the latter can differentiate to generate all cell types of the gastric mucosa (16). The powerful proliferation and tissue repair ability have made adult stem cell groups including gastric stem cells a new hotspot in research fields such as tumors and regenerative medicine (17). Based on keyword analysis, the primary study areas currently encompass surface markers, microenvironment, associated signaling pathways, and treatment resistance of GCSCs.

### 4.1 GCSCs markers

At present, the methods for identifying GCSCs in gastric cancer mainly include surface marker detection, spheroid growth experiments, and side population cell detection. Nevertheless, the intricate nature and variety of GCSCs indicators render their precise regulation mechanisms in malignancies unclear. Therefore, finding drugs that can inhibit GCSCs-related markers and their target drugs, as well as exploring new directions and ideas for the diagnosis and treatment of gastric cancer, remains a research difficulty and focus for current clinicians and scientific researchers.

With the continuous development of lineage tracing experiments, different gastric stem cell populations have been identified. These cell populations comprise villus promoters in the antrum, CCKR2 (+), Lgr5 (+), AQP5 (+) and Axin2 (+) stem cells, Mist1 (+) cells, Troy (+) and TFF2 mRNA mature chief cells in the gastric body, and Sox2, SOX9, eR1, Lrig1, and Bmi1 labeled cells in the antrum and gastric body (18). Many gastric stem cell surface markers have been proposed. These markers may not only help identify cell lineages but also provide an important basis for analyzing the plasticity of each stem cell population. Through in-depth study of these markers and gastric stem cell populations, it is expected to provide new ideas and methods for the diagnosis and treatment of gastric cancer (19).

The identification of cancer stem cells relies on markers present in ordinary stem cells, which differ based on the specific form of cancer. CD24, CD133, and CD44 are commonly present in gastrointestinal malignancies. The optimal method for identifying GCSCs is using flow cytometry to detect surface markers like CD44 and CD133. CD44 is the earliest identified and currently the most established and typical surface marker of GCSCs (20). CD44v8-10, a variant of CD44, plays an important role in gastric cancer, and its mechanism of action may be through the enhancement of antioxidant capacity. Targeted therapy for the different isoforms of CD44 and its associated molecules is presently a significant focus of research (21).

### 4.2 GCSCs and microenvironment

The development of GCSCs is not only influenced by their own changes, but also driven by cancer-associated stroma, and increasing evidence suggests that differentiated mature gastric mucosal epithelial cells can reverse differentiate into gastric stem cells under the stimulation of gastric acid, Helicobacter pylori (Hp), chronic inflammation and other factors (22, 23). Focusing on gastric stem cells and their survival environment also helps us to understand how gastric precancerous lesions are formed and what leads to cancer, which opens up a new way for cancer prevention and treatment and is one of the hot directions for research.

Hp is a gram-negative bacterium that colonizes the gastric mucosa and is an important risk factor for gastric cancer (24, 25). Hp subpopulations can penetrate deep into the gastric gland and interact directly with precursor cells, in addition to colonizing the gastric mucus and surface epithelial cells. The growth status of this gland can directly injure long-lived cells, and at the same time, this subpopulation can also trigger a massive host immune response, which further contributes to the proliferation of stem cells (26).

The hypoxic microenvironment, a fundamental trait of solid tumors, enhances tumor proliferation and dissemination. The hypoxic microenvironment triggers epithelial-mesenchymal transition, augments the stem cell-like traits of gastric cancer cells, facilitates invasion and metastasis, and manifests increased malignancy (27). Liu et al. (28) posited that exosomes from GC cells alter the gene expression and cytokine release of CD8+ T cells, thereby fostering an immunosuppressive milieu. However, studies targeting exosomes in GCSCs are relatively scarce and may be a breakthrough for future research.

### 4.3 GCSCs and gene pathways

Signaling pathways are important for maintaining normal stem cell physiology and self-renewal, and their abnormality or dysregulation is a key factor in tumorigenesis, progression and maintenance of tumor stem cell-like properties. Signaling pathways that regulate the biological properties of gastric stem cells include the Wnt, Hedgehog, Notch and Hippo signaling pathways (29–32), whilst genetic mutations in gastric stem cells frequently lead to malignant tumor development (3). For this reason, the study of gastric cancer stem cell signaling pathways and related genes is one of the hotspots of the research.

The Notch signaling system is very active in gastric cancer (GC) development and is crucial in linking GC occurrence to the proliferation of gastric stem cells. Research indicates that Notch and other signaling pathways regulate GCSCs, hence promoting tumor cell proliferation, epithelial-mesenchymal transition (EMT), metastasis, invasion, suppressing apoptosis, and tumor stromal angiogenesis (33).

The Wnt/β-catenin signaling pathway is important for stemness maintenance and proliferation of GCSCs. The non-canonical Wnt/β-catenin is an important pathway that regulates EMT and tumor stem cell formation. Liu et al. (34) showed that ICG-001 significantly inhibits the stem cell-like characteristics of GCSCs, along with the proliferation, drug resistance, and metastasis of GC cell lines, by impeding the Wnt/β-catenin signaling pathway.

Gao found that GCSCs stemness characteristics were significantly reduced after silencing or down-regulating Stearoyl-CoA-desaturase-1, which may be related to the fact that Stearoyl-CoA-desaturase-1 can activate the Hippo/YAP pathway and enhance the stemness of GCSCs (35), and studies have shown that high expression of Notum enhances gastric cancer cell stemness and up - regulates Sox2 expression via the PI3K/AKT pathway, thereby enhancing tumorigenicity of gastric cancer (36).

Recent studies have identified the FTO and SOX9 genes as being associated with GCSCs. The FTO gene, known to cause fat mass and obesity - related gene, participates in the m6A demethylation process and modulates the progression of several malignancies, including gastric cancer. The content of FTO protein in GCSCs exhibits an increasing trend. Inhibiting the FTO gene diminishes the stemness of gastric cancer cells, and inhibiting FTO may provide a viable therapeutic strategy for patients with metastatic gastric cancer (37). Chen et al. indicated that SOX9 governs the transformation of GCSCs via biased symmetric cell division (18).

### 4.4 Treatment and drug resistance of GCSCs

Conventional chemotherapy, radiotherapy and immunotherapy are commonly used to treat tumors today, but tumor recurrence and drug resistance often occur after treatment (38–40). Tumor cell resistance to chemotherapeutic agents is a major challenge in the treatment of gastrointestinal tumors, and the presence of Cancer Stem Cells in gastrointestinal tumors is an important reason. Since the exact molecular mechanism of gastric cancer stem cells is not well understood, targeted drug development and drug resistance studies remain a hot and difficult topic for future research.

Existing drug types include stem cell-targeted drugs (e.g., surface marker-targeted therapy, relevant pathway-targeted therapy, mesenchymal cell-targeted therapy, etc.), stemness-inhibiting drugs, stemness-promoting drugs, and microenvironmental regulatory drugs (41–43). GC-MSC has been verified to facilitate immune evasion and stimulate programmed death ligand 1 (PD-L1) expression in GC cells through the secretion of IL-8. Moreover, a study by He et al. further revealed that gastric cancer mesenchymal stem cells (MSCs) could augment the stemness and self-renewal capacity of gastric cancer cells via PD-L1, resulting in chemoresistance in gastric cancer (38). In addition, MSCs were shown to enhance stemness and chemoresistance in gastric cancer cells via fatty acid oxidation, both *in vitro* and *in vivo*. A new study demonstrates that MiR-21-5p augments cisplatin resistance in GCSCs by regulating the TGF-β2/SMAD signaling pathway (44). Cao et al. have found that after intervention with the Hedgehog signaling pathway inhibitor apatinib to block this pathway, the self-renewal ability of GCSCs is reduced, and they are more sensitive to chemotherapy drugs (45).

## 5 Conclusion

In summary, the current understanding of the mechanisms of GCSCs is relatively limited. More in-depth and comprehensive research is needed. On the one hand, due to the complexity and diversity of GCSCs markers, the specific regulatory mechanism in the tumorigenesis and development process is not yet completely clear. On the other hand, although researchers have made many efforts in this field, there are still many crucial problems that urgently need to be resolved. Therefore, actively searching for drugs that can inhibit GCSCs - related markers and their corresponding targets, and simultaneously exploring new directions and ideas for the diagnosis and treatment of gastric cancer have already become the key and difficult research points faced by current clinicians and scientific researchers. With advances in genomics, proteomics, and artificial intelligence and machine learning technologies, it is expected that the secrets of gastric cancer stem cells will be revealed more deeply and more specific and targeted drugs will be developed.

This paper systematically analyzes gastric cancer stem cell research using bibliometric methods and provides a theoretical basis for researchers interested in this field. However, this study has several limitations. Specifically, firstly, the data of this study were collected only from the Web of Science database, which may miss other related studies, and with the continuous updating of the database, the constant changes in the literature may have a certain impact on the amount of data. Secondly, the bibliometric analysis in this paper mainly centers on quantitative literature analysis. Although analyzing based on the number of papers represents one research perspective, there is no inherent link between quantity and quality. Thirdly, due to the wide range of collected literature, the analysis may dilute specific breakthroughs. Despite these limitations, the overall analysis in this paper still holds value in presenting the general landscape of gastric cancer stem cell research, and it can also reflect the overall trend of the research field of GCSCs, which is in the acceptable range.
