# Supplementary material for: Hotspots and trends in gastric cancer stem cell research: a visualization and bibliometric analysis
Source: Front Oncol. 2025 Mar 5;15:1523465. doi: 10.3389/fonc.2025.1523465 (PMC11919667; doi:10.3389/fonc.2025.1523465)
Supplement: Supplementary file 1 [file Table1.docx]

TABLE 1 The top 15 keywords on the research of GCSCs.

| Frequency Ranking | | | |  | Centrality Ranking | | | |
| --- | --- | --- | --- | --- | --- | --- | --- | --- |
| Count | Central | Year | keyword | Rank | Central | Count | Year | keyword |
| 1779 | 0.71 | 1996 | gastric cancer | 1 | 0.71 | 1779 | 1996 | gastric cancer |
| 1178 | 0.2 | 1994 | stem cells | 2 | 0.36 | 146 | 1998 | lung cancer |
| 995 | 0.14 | 1994 | expression | 3 | 0.26 | 302 | 1994 | carcnoma |
| 490 | 0.01 | 2007 | cancer stem cells | 4 | 0.25 | 287 | 1994 | cancer |
| 409 | 0.11 | 1997 | breast cancer | 5 | 0.2 | 1178 | 1994 | stem cells |
| 387 | 0.08 | 2002 | identification | 6 | 0.14 | 995 | 1994 | expression |
| 326 | 0.03 | 2003 | colorectal cancer | 7 | 0.14 | 222 | 2000 | helicobacter pylori |
| 302 | 0.26 | 1994 | carcnoma | 8 | 0.14 | 128 | 1999 | protein |
| 301 | 0.04 | 2008 | metastasis | 9 | 0.12 | 198 | 2001 | ditterentiation |
| 299 | 0.02 | 2009 | epithelial mesenchymal transition | 10 | 0.11 | 409 | 1997 | breast cancer |
| 287 | 0.25 | 1994 | cancer | 11 | 0.11 | 206 | 2000 | gene expression |
| 278 | 0.03 | 2003 | proliferation | 12 | 0.11 | 193 | 2002 | gene |
| 254 | 0.1 | 2006 | growth | 13 | 0.11 | 104 | 2005 | up requlation |
| 222 | 0.14 | 2000 | helicobacter pylori | 14 | 0.1 | 254 | 2006 | growth |
| 210 | 0.02 | 2006 | in vitro | 15 | 0.08 | 387 | 2002 | identification |

TABLE 2 The top 10 co-cited references on the research of GCSCS.

| Rank | Cited reference | Journal | citations |
| --- | --- | --- | --- |
| 1 | Identification of gastric cancer stem cells using the cell surface marker CD44 | Stem Cells | 380 |
| 2 | Lgr5(+ve) stem cells drive self-renewal in the stomach and build long-lived gastric units in vitro | Cell Stem Cell | 279 |
| 3 | Global cancer statistics | CA Cancer J Clin | 279 |
| 4 | Prospective identification of tumorigenic breast cancer cells | Proc Natl Acad Sci USA | 211 |
| 5 | Stem cells, cancer, and cancer stem cells | Nature | 206 |
| 6 | Comprehensive molecular characterization of gastric adenocarcinoma | Nature | 187 |
| 7 | The epithelial-mesenchymal transition generates cells with properties of stem cells | Cell | 168 |
| 8 | Identification of stem cells in small intestine and colon by marker gene Lgr5 | Nature | 158 |
| 9 | Gastric cancer originating from bone marrow-derived cells | Science | 158 |
| 10 | The two histological main types of gastric carcinoma: diffuse and so-called intestinal-type carcinoma. An attempt at a histo-clinical classification | Acta Pathol Microbiol Scand | 154 |
